# Neurobiological mechanisms and treatment of empathic pain in nonsuicidal self-injury

**DOI:** 10.3389/fpsyt.2026.1681883

**Published:** 2026-01-30

**Authors:** Shu-Li Xu, Di Wu, Min Cai, Jing-Wen Li, Long-Biao Cui, Wen-Jun Wu

**Affiliations:** 1 Department of Psychiatry, Xijing Hospital, Fourth Military Medical University, Xi’an, China; 2 Department of Psychiatry, 986 Hospital, Fourth Military Medical University, Xi’an, China; 3 Shaanxi Provincial Key Laboratory of Clinic Genetics, Fourth Military Medical University, Xi’an, China

**Keywords:** anterior cingulate cortex (ACC), empathic pain, neural network, neurobiology, nonsuicidal self-injury

## Abstract

Empathic pain is defined as the experience of vicarious pain resulting from the observation of another person’s suffering, and it involves complex neurobiological pathways that parallel those involved in direct pain perception. The intricate link between nonsuicidal self-injury (NSSI) and empathic pain lies in the fact that they share certain neurobiological mechanisms, particularly in the areas of emotion regulation and pain perception, and both phenomena involve brain networks involved in pain processing, emotion regulation, and social cognition. This review examines the current understanding of these mechanisms, including cellular and molecular pathways, changes in neural networks, and factors that influence the development of empathic pain. It also explores potential therapeutic strategies to address the complex interactions between empathic pain and NSSI, focusing on psychological interventions, pharmacological approaches, and novel neurostimulation techniques.

## 1 Introduction

Nonsuicidal self‐injury (NSSI) refers to the act of deliberately destroying one’s own body tissue without conscious intent to die and for reasons that are not socially sanctioned (1). The drivers of this behavioral pattern are complex, including not only psychosocial factors but also neurobiological mechanisms. In recent years, researchers have begun to notice a potential association between NSSI and empathic pain, which is the painful feelings that individuals experience when they witness or visualize another person suffering (2). Not only is empathic pain a core component of the human capacity for empathy, but in some cases, it may also be an important factor in triggering or exacerbating NSSI behaviors (3). Individuals who are prone to nonsuicidal self-harm may exhibit stronger empathic pain responses, possibly because of their shared weaknesses in emotion regulation and neural processing (4). Understanding these neurobiological overlaps is critical for developing targeted treatments.

Neurobiological studies reveal that empathic pain and NSSI share a series of common brain networks, particularly those involved in pain processing, emotion regulation, and social cognition (5). For example, the anterior cingulate cortex, insula, and mirror neuron system play key roles in the neural representations of empathic pain and NSSI (6). In addition, changes in the activity of the endogenous opioid system and the dopamine pathway have been identified as foundational neurochemical components mediating the link between the two (7). Given the neurobiological intersection between empathic pain and NSSI, the development of targeted treatment strategies is particularly urgent. Traditional psychotherapeutic approaches, such as cognitive–behavioral therapy, dialectical behavioral therapy, and mindfulness meditation, have been shown to be highly effective in helping individuals improve their emotional regulation and alleviate feelings of pain (8). However, given the specificity of empathic pain, integrating social skills training, enhancing the quality of interpersonal interactions, and employing neuroscience-oriented interventions, such as neurofeedback and transcranial magnetic stimulation, are emerging as new directions in the treatment of NSSI (9). The aim of this review is to delve into the neurobiological mechanisms of empathic pain in NSSI, analyze its impact on individual behavior, and propose an integrated treatment approach based on latest research.

## 2 Cellular and molecular mechanisms of empathic pain in NSSI

The cellular and molecular mechanisms involved in the relationship between NSSI and empathic pain involve complex interactions among multiple systems and pathways in the brain, such as primary afferent nerve fibers, neurotransmitters and peptides, the endogenous opioid system, the mirror neuron system, neurotrophic factors and cellular stress (10). A number of key cellular and molecular mechanisms play important roles in these processes. Pain signals are first captured by injury receptors in the skin, muscles, and joints, which send signals through primary afferent nerve fibers to the spinal cord and then to the brain (11). During pain transmission, a variety of neurotransmitters and peptides are released, including substance P, calcitonin gene-related peptide, bradykinin, and they are involved in the amplification and modulation of pain signals (12). In pain and emotion regulation, the release of β-endorphin acts as an analgesic and emotional calming agent, and in NSSI, self-injury may trigger this system to produce a transient sense of relief (13). When an individual observes another person in pain, mirror neurons are activated, enabling the observer to mimic and experience a similar sensation, helping the individual experience and understand the feelings of the other person, and causes them to feel similar pain; this may underlie empathic pain (14). Neurotrophic factors, such as brain-derived neurotrophic factor, play key roles in neuronal growth, survival, and function; chronic stress and mood disorders may affect the levels of these factors, which in turn may affect pain perception and mood regulation; inflammatory states may affect pain thresholds and mood states, and inflammatory mediators, such as cytokines (TNF-alpha, IL-1beta, and IL-6) and oxidative stress products, may be involved (15).

NSSI is a complex behavior involving multiple mechanisms and motivations, and cannot be explained by a single hypothesis. The endogenous opioid substance hypothesis is a very core neurobiological hypothesis. This hypothesis suggests that some individuals with NSSI may have a low baseline level of the endogenous opioid system, manifesting as emotional pain, anxiety, emptiness, and numbness (16). The intense pain and stress caused by self-harm behavior trigger the brain to release a large amount of endogenous opioid substances, such as endorphins, which instantly produce a brief but intense sense of pleasure and relieve emotional pain (17). Some studies have shown that individuals with NSSI report feelings of calm or relief after self-harm, and administering opioid antagonists can, to a certain extent, reduce the self-harm impulses of some individuals (18). However, not all individuals are like this. This mechanism may be more applicable to those who mainly have the motivation to relieve intense negative emotions, while for those with NSSI whose main motivation is self-punishment, the opioid mechanism may not be dominant (19). The relationship between empathy pain and NSSI may not be a direct causal relationship, but rather the result of the combined effects of shared cellular and molecular mechanism disorders, neural circuit dysregulation, and emotional regulation failure. An imbalanced empathy pain system makes individuals more prone to being troubled by external pain, while a collapsed emotional regulation system drives them to respond with their own pain. Empathy pain serves as an emotional trigger point, such as witnessing or imagining the pain of others. For NSSI individuals who are already emotionally vulnerable, this may be a strong negative emotional stimulus. When they are unable to regulate the pain caused by empathy in a healthy way, they may use their own pain to divert and cover the unbearable emotional pain.

## 3 Changes in empathic pain neural networks during NSSI

NSSI and empathic pain involve changes in neural networks in multiple regions of the brain that reflect the complex interactions of pain perception networks, emotion regulation networks, and social cognitive networks, as outlined below in relation to NSSI and empathic pain. The primary and secondary sensory cortex in the pain perception network processes the physical properties of pain, and the anterior cingulate cortex (ACC) plays a key role in pain assessment and emotion regulation and is closely related to the subjective perception of pain; these brain regions are involved in pain processing and emotional responses (20). Regarding empathic pain, increased activity in these regions reflects the individual’s perception of pain in others. Similarly, in NSSI, abnormal activity in these regions may be associated with abnormal modulation of pain. The insula is involved in the visceral and emotional components of pain, as well as pain anticipation and memory (21). The emotion regulation system includes the prefrontal cortex, amygdala, and hippocampus; the prefrontal cortex, particularly the ventral and dorsolateral prefrontal cortex, is responsible for higher cognitive functions such as decision-making, planning, and inhibitory control, which are critical for emotion regulation (22). The amygdala plays a central role in emotional processing and fear responses and may be involved in the negative emotional experience of pain (23); the hippocampus is associated with the formation and storage of emotional memories and may influence long-term memory and anticipation of pain (24); repetitive NSSI behaviors may activate the dopamine reward pathway in the brain, similar to an addictive process that produces immediate emotional relief (25); and the striatum is associated with habit formation and motor control and may be involved in the automation of NSSI behaviors (26). In addition, empathic pain causes changes in social cognitive networks: the mirror neuron system, located in the inferior frontal gyrus, parietal lobe, and premotor cortex, is involved in observing and mimicking the behaviors of others and is critical to the experience of empathic pain (27); the temporoparietal junction plays a role in theorizing about the mental states of others and the ability to empathize, potentially affecting the intensity and experience of empathic pain (28); and the default mode network, which is involved in the simulation of self-reflection and social contexts and may play a role in understanding the association between others’ pain and the self-perception of pain (29). In the relationship between NSSI and empathic pain, there are complex interactions between the above networks. For example, empathic pain may affect the emotion regulation network by activating the pain perception network and the social cognition network, leading individuals to be more likely to adopt self-injurious behaviors as a coping mechanism when facing emotional stress.

## 4 Electroencephalogram/event-related potentials researches of empathic pain in NSSI

EEG/ERP researches help us to understand the temporal dynamics of empathic processing. There has been a study that asked participants to watch images with facial expressions and hear verbal descriptions of pain before the images. The ERP research found that emotional intonation might interact with facial expressions and semantic content, suggesting that its emotional intonation has the function of a lateral communication signal (30). Social relationships and emotional relationships may affect empathy for others’ emotional states. Some studies have asked participants to complete pain decision-making tasks involving upright and inverted facial stimuli (pain or neutral stimuli), and through ERP technology to monitor the neural empathy responses of the subjects, it was found that the perceived physical distance might affect empathy, as well as social and emotional distance (31). Another ERP study found that the areas surrounding the human body have a crucial impact on the interaction between the individual and the environment. Physical barriers reduce the cortical activation of regions in the brain that regulate interpersonal interaction (i.e., the primary cortex, somatosensory cortex, premotor cortex, and inferior frontal gyrus), preventing the possibility of interaction and thus reducing the observer’s empathy (32). Additionally, models of how cognitive architectures process the perception and cognitive information of others’ mental states are usually described as a two-layer model, considering two independent systems. In the context of neurosciences regarding empathy, ERP technology has discovered that the neural pathways for the perception and cognitive processing of pain are different, at least when the cognitive components are triggered by language (33). Empathic excitement is an early constituent of empathic ability that appears in infancy and early childhood. EEG event-related potential studies provide neurophysiological evidence for the formation of empathy in childhood, as emotional responses gradually weaken with age while cognitive assessments strengthen. As the developmental process progresses, empathic excitement is increasingly associated with the ability to distinguish between oneself and others, enabling people to better regulate contagious painful emotions (34).

EEG/ERP studies are helpful for us to understand the processing patterns, neural development and cognitive theories of empathic pain, and can also further elucidate the neurobiological mechanisms of empathic pain in non-suicidal self-injury (35). The EEG/ERP changes have deepened our understanding of the emotional regulation dysfunction in NSSI, indicating that the problem lies not only in the processing of one’s own emotions, but also in the processing of social emotional signals. This provides crucial neuroscientific evidence for us to understand their complex inner emotional world. These characteristics may serve as objective biomarkers for identifying the risk of NSSI and evaluating the effectiveness of intervention, helping patients adjust their early vigilance and late avoidance patterns to social pain signals, and establishing healthier empathy and emotional regulation strategies.

## 5 Factors affecting the development of empathic pain in NSSI

Empathy, as a bond of social connection, from an evolutionary perspective, the sharing of this neural mechanism forms the basis of altruistic behavior and social connection. By feeling the pain of others, we can generate the motivation to help and comfort, thereby promoting cooperation and social cohesion (36). Empathic pain refers to the neural networks related to pain that are activated in our own brains when we see others suffering. Somatic pain and empathic pain have overlapping neural networks, which bring psychological connections. However, this connection is regulated by various factors, allowing us to empathize while not being completely overwhelmed by the pain of others (37). A healthy brain can clearly distinguish between one’s own and others’ pain. This is achieved through another area in the brain called the default mode network, especially the medial prefrontal cortex, which is more active when thinking about others’ states, helping us adopt perspectives and understand that this is the pain of others rather than our own (38). Only when the distinction between self and others fails, empathic pain may transform into personal pain. At this time, individuals may excessively immerse themselves in the negative emotions triggered by the pain of others, which may lead to empathic avoidance or empathic exhaustion rather than generating altruistic behavior.

The development of empathic pain in NSSI is influenced by a variety of factors that involve both internal traits of the individual and external environmental conditions. The key factors influencing the development of empathic pain in NSSI may include personal factors, mental health conditions, socioenvironmental factors, and biological factors.

An individual’s genetic background may influence his or her pain sensitivity and emotional regulation and thus his or her experience and expression of empathic pain (39). Individuals with personality traits such as high neuroticism, low emotional stability, high sensitivity, and low impulse control may be more likely to experience intense empathic pain and may turn to NSSI as a coping mechanism as a result (40). The lack of effective emotion regulation strategies may make it difficult for individuals to deal with negative emotions triggered by empathic pain, thereby increasing the risk of NSSI. Additionally, highly empathic individuals may be more sensitive to the pain of others, which may increase their experience of empathic pain, especially in the absence of appropriate emotion regulation strategies (41). Mental health conditions can influence the development of empathic pain in NSSI, e.g., individuals with depression or anxiety disorders may be more inclined to experience empathic pain because of their greater sensitivity to emotional stimuli; individuals who have experienced a traumatic event may be more likely to trigger a strong empathic response when they see or think of another person’s pain (42); borderline personality disorders may exhibit unstable emotional responses and interpersonal relationships, increasing empathy toward pain and subsequent NSSI behaviors (43). Socioenvironmental factors also influence the development of empathic pain in NSSI, such as family conflict, neglect, or abuse, which may increase an individual’s pain sensitivity and tendency to empathize with pain, as well as promote NSSI as a way to cope with family stressors (44). Self-injurious behaviors observed among peers may increase an individual’s risk of NSSI through a mimicry effect, while the level of peer support may influence an individual’s processing of empathic pain (45). One study modeled the role of perceptual empathy and found that empathy may arise from mapping another person’s state to one’s own nervous system and that when individuals are frequently exposed to images or stories depicting another person’s suffering, such content may stimulate or exacerbate empathic pain (46). The neuroendocrine system plays an important role in empathic pain, e.g., levels of stress hormones such as cortisol may affect an individual’s perception of pain and regulation of mood, which in turn affects empathic pain and NSSI behavior (47). Neurotransmitter imbalances, such as imbalances in serotonin, dopamine, and norepinephrine, may affect emotion regulation and pain processing, increasing the experience of empathic pain (48).

The relationship between physical pain and empathic pain is both close and complex. Research has found that when a person experiences their own pain and sees the pain of others, the neural network known as the pain matrix in the brain is activated, including the anterior cingulate cortex (ACC), especially the dorsal part, which mainly processes the motivational components of pain, namely the unpleasant and uncomfortable feelings brought by pain; the anterior insula is responsible for processing the internal sensation components of pain, integrating physical sensations with emotional states, allowing us to feel pain; the primary and secondary somatosensory cortices mainly process the sensory discrimination components of pain (49). The core areas where physical pain and empathic pain overlap to a high degree mainly include the anterior cingulate cortex and the anterior insula, which enables us to empathize with others’ suffering to some extent, activating the emotional centers related to our own pain. However, physical pain strongly activates the somatosensory cortex because it needs to process real, physical signals from the body; while in empathic pain, the activation of the somatosensory cortex is usually weak, indicating that although we can feel others’ discomfort, we do not truly experience the same stabbing or burning sensation in our bodies. In individuals with NSSI or other long-term psychological traumas, their ability to empathize with others’ pain may be dysregulated, either being overly sensitive to the point of being unbearable, or being overly inhibited to the point of feeling isolated from others (50). Both of these situations reflect the impairment of their core emotional regulation functions. The relationship between physical pain and empathic pain may show abnormalities. They may experience numbness in their own pain emotional experiences due to long-term emotional pain, and therefore use physical pain to awaken themselves or regulate their emotions, resulting in self-harm behaviors (51).

## 6 Treatment strategies for empathic pain in NSSI

Empathic pain and NSSI share molecular biological pathways and neural circuits. In individuals with NSSI, they may become numb to their own pain emotions due to long-term emotional distress, and thus use physical pain to awaken themselves or regulate their emotions, resulting in self-harm behaviors (52). Early identification of abnormal empathic pain and understanding the neurobiological mechanisms of empathic pain and NSSI are helpful in preventing and treating NSSI. Incorporating pain empathy into the treatment goals represents a shift from traditional, isolated symptom management to a more holistic, relational, and neurobiological treatment paradigm, which is of great significance for the treatment of NSSI. Individuals with NSSI may trigger emotions they cannot bear due to excessive empathy for others, and then use self-harm to regulate. The treatment based on empathy actually trains the brain’s more fundamental regulatory and discrimination functions. Dysregulation of empathy ability is the root cause of many interpersonal conflicts and social difficulties. Calibrating empathy ability can significantly improve social functions (53). Incorporating pain empathy into the treatment goals means that we recognize that humans are social beings, and our brains are naturally interconnected and capable of feeling. Treatment is no longer simply eliminating symptoms, but helping patients master a core interpersonal survival skill, how to reasonably use empathy, so as to establish deep connections with others while protecting their inner world from being consumed, and ultimately achieving a healthy balance that is both caring and bounded (54).

The following are some possible directions for treatment:

A first treatment is to help individuals identify and change the cognitive patterns that lead to self-injurious behaviors and learn healthier coping strategies, such as emotion regulation skills, problem-solving approaches, and interpersonal skills. Dialectical behavior therapy (DBT) is particularly appropriate for individuals who have difficulty regulating emotions and strong emotional reactions. Cognitive behavioral therapy (CBT) emphasizes positive thinking, emotion regulation, pain tolerance, and interpersonal effectiveness skills (55). Empathy-focused therapy in the context of empathic pain can help individuals understand and process empathic responses with their therapist or others, promoting healthier attachment patterns and relationships (56). Psychodynamic therapy can explore the impact of an individual’s subconscious psychological conflicts and childhood experiences on pain, helping the individual understand the psychological reasons behind the pain (57). Positive thinking meditation can help individuals learn to observe their emotions and physical sensations without reacting immediately, thus reducing impulsive self-injurious behavior (58). Providing information about the psychology of pain, empathic mechanisms, and self-injurious behaviors can help individuals and families understand the nature of these issues, reduce shame and misunderstanding, and facilitate the healing process. Helping individuals build and maintain healthy relationships and improve their social skills can reduce empathic pain and self-injurious impulses that arise from loneliness and social conflict.

Second, although pharmacotherapy is directed primarily at concomitant psychological disorders such as depression and anxiety, in some cases, medications can complement psychotherapy, such as the use of antidepressants or mood stabilizers to improve mood regulation. Using opioid receptor antagonists or agonists can modulate the endogenous opioid system, and inhibit inflammatory mediators to reduce the effects of inflammation on empathic pain (59). Selective serotonin reuptake inhibitors (SSRIs) work by blocking the reuptake of serotonin by presynaptic neurons, thereby increasing the concentration of serotonin in the synaptic cleft, thus enhancing serotonergic neural transmission. The serotonin system plays a central role in regulating emotions, social behaviors, and pain perception. The effects of SSRIs can be summarized as emotional relief and cognitive clarity. They mainly reduce the excessive activity in brain regions related to personal pain (such as the ACC and anterior insula) to decrease the uncomfortable, self-centered emotional components in empathy. This enables individuals to understand and respond to others’ pain more effectively without excessive emotional involvement. The use of selective serotonin reuptake inhibitors (e.g., SSRIs) can modulate the levels of neurotrophic factors (60).

Third, with advances in neuroscience techniques, such as functional magnetic resonance imaging, transcranial magnetic stimulation, and electrophysiological recordings, researchers have been able to explore the dynamics of these neural networks in NSSI and empathic pain in greater depth, opening the possibility of developing more precise diagnostic tools and treatment options (61). Repetitive transcranial magnetic stimulation (rTMS) is a non-invasive brain stimulation technique that uses changing magnetic fields to induce weak currents in specific areas of the cerebral cortex, thereby regulating the excitability of neurons. Although rTMS mainly acts on the cortical regions, its effects can be transmitted through the neural network to deeper structures connected to the target area, such as the anterior cingulate cortex. The rTMS can stimulate the prefrontal cortex regions that have a close functional connection with the ACC, especially the dorsolateral prefrontal cortex, to change the excitability of neurons in the target area and the connected network (62). The rTMS mainly functions by regulating the balance of the large brain network where the ACC is located (such as the frontal-limbic system), which may be particularly effective for improving emotional regulation and excessive emotional responses in empathic pain. Precision modulation strategies targeting mirror neuron systems and related neural pathways, such as transcranial magnetic stimulation and deep farcical stimulation, can modulate the activity of brain regions associated with pain and emotion and may represent a new direction in the treatment of empathic pain (63). However, research in these areas is still ongoing, and future work will aim to more fully understand the details of these neural networks and their role in individual differences and treatment response.

Whether through physical regulation (rTMS) or chemical regulation (SSRIs), the ultimate goal is to restore the balance of the frontal-limbic system network, particularly to regulate the abnormal activities of the ACC in emotion and pain processing. For individuals with abnormal empathy-based pain processing who engage in non-suicidal self-harm, both of these methods offer promising approaches to alleviate symptoms and promote recovery by altering the underlying neural circuits. In the future, precision medicine may combine biomarkers such as electroencephalography and fMRI to help patients choose the most suitable intervention method.

In addition, regular physical exercise and a healthy lifestyle not only are beneficial to physical health but can also improve mood, help individuals vent bad feelings, and reduce their tendency toward self-injury. Participation in a group consisting of people experiencing similar challenges can provide emotional support and practical advice, reduce isolation and enhance therapeutic effects. The participation and support of family members is very important to the therapeutic process. Family therapy can help improve family communication, resolve potential family conflicts and create a more supportive environment (64). For individuals in crisis, emergency crisis intervention services are necessary to ensure safety and provide immediate psychological support (65). Ongoing follow-up and monitoring are important to prevent recurrence, and regular evaluation and adjustment of the treatment plan can help maintain the effectiveness of the treatment.

Each individual’s situation is unique, so treatment plans should be individualized and tailored to the person’s specific needs and circumstances. The treatment process requires patience and time, and it is important to maintain open lines of communication and adjust strategies as appropriate based on treatment progress. The goal of treating NSSI and empathic pain is to help individuals develop healthy coping mechanisms and reduce self-injurious behaviors while enhancing their emotional regulation and social functioning. Because the problem of empathic pain in NSSI is multifaceted, a comprehensive treatment program is often the most effective.

**Figure 1:**
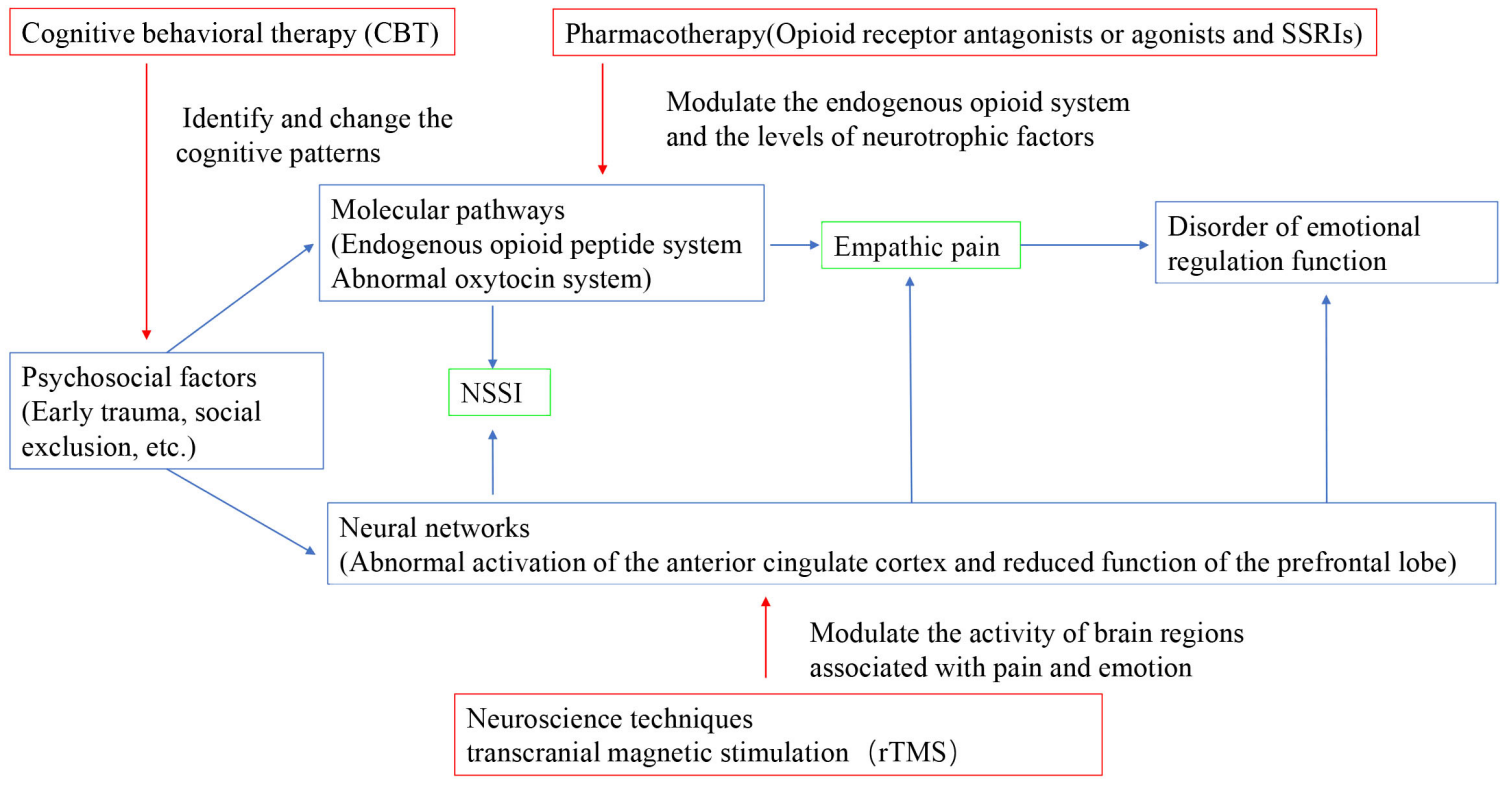
The mechanisms and therapies interact with each other in the context of empathic pain and NSSI.

Psychosocial risk factors (especially early trauma) shape a dysfunctional emotional regulation system (the core mechanism), which manifests biologically as functional abnormalities at the molecular level (opioid and oxytocin systems) and at the neural network level (pain matrix and regulatory networks). These biological abnormalities collectively lead to two seemingly different but intrinsically related behavioral manifestations: using NSSI to cope with unbearable internal pain; and, when facing the pain of others, experiencing abnormal empathetic responses (either excessive involvement and pain, or emotional numbness and detachment). The NSSI behavior and the abnormal empathetic responses will, in turn, reinforce the difficulty in emotional regulation and may exacerbate social isolation, thus forming a vicious cycle. Therefore, NSSI and empathic pain are not two independent issues; they share a common, dysregulated pain perception and regulation system. Understanding this connection is of great significance for developing neurobiological markers for NSSI and more effective psychological/medicinal intervention methods (such as psychological therapies targeting the opioid system or empathy training). **Figure 1** illustrated the mechanisms and therapies interact with each other in the context of empathic pain and NSSI.

## 7 The direct relationship between empathic pain and NSSI

Empathic pain refers to the phenomenon in which an individual truly feels pain or discomfort when observing others experiencing pain. It is an extreme and embodied form of pain empathy. There is a direct and seemingly contradictory relationship between it and NSSI, which is mainly manifested as an immediate coping mechanism of using pain to control pain.

Frequent and high-intensity involvement in the pain of others can also lead to long-term emotional and sensory overload, causing individuals to be in a state of empathetic stress for a long time. This chronic stress can lead to anxiety, tension, and emotional exhaustion. In this case, NSSI is no longer intended to terminate a specific instance of empathetic pain, but rather serves as the ultimate means of regulating this chronic overload state. When negative emotions accumulated through empathy reach an unbearable critical point, NSSI helps individuals awaken themselves from emotional numbness, break free from dissociation or overload, focus attention on controllable physical pain, and achieve rapid physiological relaxation, such as through the release of endogenous opioids (66).

For some individuals, when they witness or imagine the pain of others, their brains generate a real and tangible sense of pain, which may be related to overactive mirror neuron systems and strong activation of related brain regions, such as the anterior insula and anterior cingulate cortex (67). Their brains are unable to effectively distinguish between ‘other people’s pain’ and ‘their own pain’. This empathetic pain itself becomes a strong and undeniable negative sensory stimulus. When this feeling becomes unbearable, individuals will urgently need to make it stop. At this point, NSSI is used as a direct tool to interrupt the empathetic, uncontrolled pain signal by applying a stronger and more controllable self-pain. The internal pain caused by empathy itself, whether it is specific pain or diffuse pressure, directly drives individuals to adopt NSSI behavior as a rapid and pathological self-regulation tool, in order to immediately terminate or escape this unbearable state.

It is worth noting that long-term NSSI behavior can lead to a blunting of self-pain perception and an increase in pain threshold by (68). This is in stark contrast to ‘empathetic pain’ and forms a vicious cycle: individuals suffer due to their high sensitivity to the pain of others (empathetic pain), and in order to alleviate this pain, they adopt NSSI. Repeated NSSI caused him to become desensitized to his own pain. Pain dullness makes it physiologically easier to implement NSSI, allowing behavior to be maintained and exacerbated. This further solidifies NSSI as the primary way to cope with empathetic pain.

**Figure 2:**
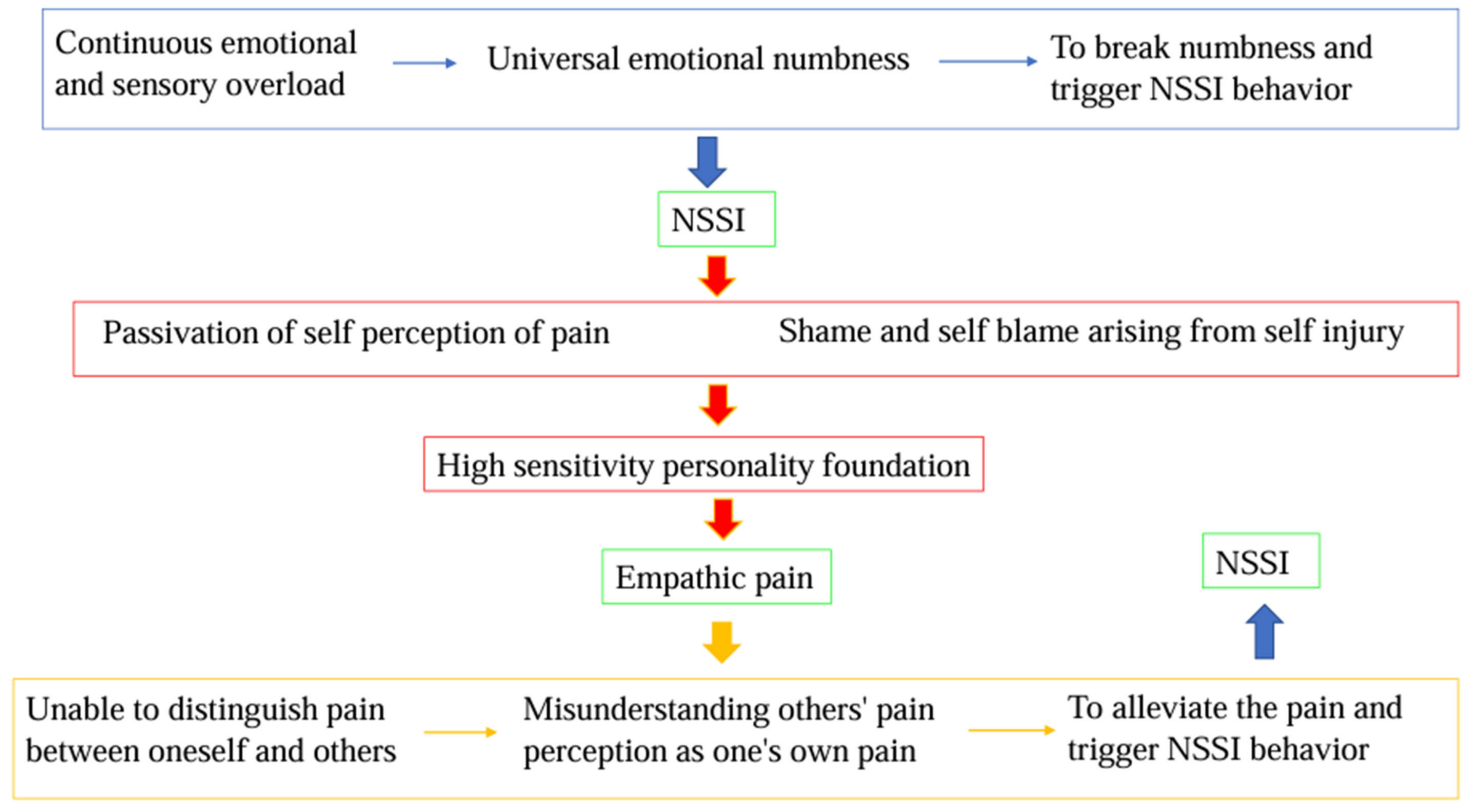
A reinforcing vicious cycle is formed between empathetic pain and NSSI.

That is to say, for some individuals, empathetic pain itself is a direct and acute trigger for NSSI. For more people, chronic emotional overload caused by the accumulation of empathetic pain is a core psychological pathological mechanism driving NSSI behavior. The paradoxical combination of pain dullness after NSSI and previously high sensitivity to empathetic pain jointly maintains this behavior. Understanding this relationship has important clinical significance, as it can help NSSI patients identify and manage their excessive empathic responses, establish healthy emotional boundaries, cope with sensory and emotional discomfort caused by empathy, and thus break this dangerous cycle. **Figure 2** illustrated that a reinforcing vicious cycle is formed between empathetic pain and NSSI.

## 8 Limitations and prospects

The literature included in this review reflects that the operational definition of NSSI remains somewhat ambiguous in the academic field. The behaviors constituting NSSI exhibit significant variability in manifestations, functional intentions, severity, and medical harm. However, most neurobiological studies have not conducted fine-grained distinctions among these heterogeneous subgroups. Combining groups with vastly different motivations and manifestations for analysis may result in obscuring more precise mechanisms within specific subgroups—undoubtedly complicating the derivation of universal conclusions. Moreover, the neurobiological mechanisms discussed in this review are primarily inferred from indirect evidence, including functional magnetic resonance imaging (fMRI), hormonal level measurements, and genetic association studies. Each of these methods has its limitations: fMRI’s correlational results cannot establish causality; peripheral biomarkers (e.g., cortisol) struggle to fully reflect real-time activity in the central nervous system. More critically, current findings still fail to clearly distinguish whether certain neurobiological features are predisposing factors for NSSI behavior, consequences of repeated behaviors, or manifestations of broader mental comorbidities (e.g., depression, borderline personality disorder). Consequently, the mechanistic models proposed in this review remain inherently speculative and require further validation and clarification through more refined experimental designs such as longitudinal tracking and causal intervention studies.

The research on non-suicidal self-injury and empathic pain is a rapidly developing but still immature field. There are many limitations in terms of methodology, theory and mechanism, as well as research methods. Firstly, most studies use standardized static images or short videos to induce empathic pain, which is far from the complex, dynamic and context-rich pain expressions in real life and cannot capture the complexity of how NSSI individuals process others’ emotional signals in real social situations. Secondly, most current electroencephalogram (EEG) studies are cross-sectional, which can only reveal the correlation between NSSI and brain responses to empathic pain, but cannot determine the causal relationship1. For example, is the NSSI behavior causing the abnormal empathy, or does the abnormal empathy predispose the individual to engage in NSSI, or are both caused by other factors such as childhood trauma? Therefore, there are limitations in terms of correlation and causality, and there is a lack of longitudinal studies. In addition, measurement tools have limitations. EEG/ERP, although having high temporal resolution, has insufficient spatial resolution and is weak in precisely locating the source of brain activity (69). Moreover, there may be inconsistency between individual subjective empathy reports and their neural responses, and the measurement indicators of empathic pain are also diverse (70).

There are also limitations in terms of theory and mechanism. There is a high degree of heterogeneity within the NSSI group. Different self-harm functions (such as to regulate emotions, to punish oneself, to feel something), different methods (cutting vs. hitting), and different comorbidities (depression, borderline personality disorder, autism spectrum) may correspond to completely different coping patterns for empathic pain. However, existing studies often treat them as a homogeneous group for processing, ignoring the heterogeneity. Moreover, most studies target adolescent or adult populations, lacking a developmental perspective (71). However, NSSI typically begins during adolescence, which is a critical period for brain development and the development of empathy skills. Currently, little is known about how the development trajectory of the neural circuits for empathic pain interacts with the emergence and maintenance of NSSI.

Then we will discuss the limitations of the samples and the controls. Many studies use healthy control groups, but a more rigorous design requires the establishment of clinical control groups to determine whether the observed effects are specific to NSSI or are common to all emotional disorders (72). Additionally, it is important to be aware of the confounding factors of comorbidity and medications (73). Individuals with NSSI often have other mental disorders and take psychotropic medications, and these factors themselves can affect EEG/ERP signals and empathic responses (74). It is difficult to completely control or separate their effects in the research.

Based on these limitations, future research can make breakthroughs in multiple directions such as technological innovation, deepening of theories and mechanisms, and the transformation of clinical applications. In terms of technological innovation, virtual reality technology can be utilized to create immersive and interactive social pain scenarios, using more personalized stimuli to enhance ecological validity. Combined with high temporal resolution EEG/ERP and high spatial resolution fMRI, multimodal brain imaging fusion can be adopted to construct the trajectory of empathic pain processing in time and space, and precisely depict the complete brain network dynamics from early perception to late evaluation. In terms of the deepening of theories and mechanisms, the heterogeneity of NSSI can be analyzed, and subgroup analysis can be consciously included in the research design (75). Participants can be subdivided based on the functions, frequencies, severity, and comorbid conditions of NSSI, and neurobiological markers related to specific clinical symptoms can be sought. Longitudinal tracking studies can also be increased to observe how the neural indicators of empathic pain change over time in high-risk adolescent groups (such as those with childhood trauma or emotional distress), and which indicators can predict the onset and outcome of NSSI (76). Additionally, most current studies are small-sample studies. In future research, it is necessary to expand the sample size, establish a database of empathic pain in NSSI, and supplement factors such as gender, age, cultural differences, and comorbid emotional disorders for the influence of NSSI on empathic pain (77).

In terms of theories and mechanisms, neuroregulatory techniques such as rTMS can be used as experimental tools to actively regulate specific brain regions such as ACC, observing their effects on empathic pain behaviors and related neural activities, to verify causal hypotheses. Combining genetics and pharmacology, the effects of related gene polymorphisms or drug manipulation on the empathic pain responses of NSSI individuals can be examined, and the molecular mechanisms of empathic pain in NSSI can be explored.

Finally, the clinical application of research results should be transformed in the future, exploring whether specific EEG/ERP patterns can serve as objective biomarkers for identifying NSSI risks, assisting in diagnosis or classifying subtypes, and developing predictive and diagnostic tools. Before and after psychological or pharmacological treatment, the neural indicators of empathic pain in patients can be measured to see if they normalize with symptom improvement, which can serve as a sensitive indicator for evaluating treatment effectiveness, assessing treatment response and prognosis, and achieving precise mental medical intervention based on the patient’s neural characteristics, guiding personalized treatment. In summary, the research on empathic pain in NSSI is standing at a turning point from descriptive phenomena to elucidation of mechanisms. Future research needs to adopt more refined designs, more advanced technologies, and more developed perspectives to predict, intervene and prevent NSSI.

In NSSI, individuals may self-injure as a means of emotion regulation to alleviate internalized emotional distress. Empathic pain may exacerbate this need, as the observation of another person’s distress may trigger an individual’s own emotional distress, prompting them to seek immediate relief, including self-injurious behaviors. Empathic pain in NSSI is a complex phenomenon that involves multiple facets and hides fine-grained cellular and molecular mechanisms, complex neural network changes, and a developmental process that is influenced by multiple factors. Understanding these cellular and molecular mechanisms is critical for the development of novel therapeutic strategies targeting NSSI and empathic pain. By intervening in these pathways, it is possible to reduce pain perception, improve emotion regulation, reduce self-injurious behaviors, and enhance an individual’s social functioning and quality of life. By further unraveling these mechanisms and developing targeted interventions, we will not only be able to better understand the nature of human empathy but also be able to provide effective help and support to individuals who suffer from excessive empathy. Future research should continue to explore these areas with the aim of developing more personalized and efficient treatment strategies. By improving our understanding of the complex phenomenon of empathic pain in NSSI, we expect to provide new perspectives and strategies for clinical practice to more effectively support individuals who are suffering from NSSI.
